# Deconvolution of gene expression from cell populations across the *C*. *elegans* lineage

**DOI:** 10.1186/1471-2105-14-204

**Published:** 2013-06-22

**Authors:** Joshua T Burdick, John Isaac Murray

**Affiliations:** 1 Genomics and Computational Biology Group, University of Pennsylvania, 440 Clinical Research Building 415 Curie Boulevard, Philadelphia, PA 19104; 2 Department of Genetics, Perelman School of Medicine, University of Pennsylvania, 437A Clinical Research Building, 415 Curie Boulevard, Philadelphia, PA 19104

## Abstract

**Background:**

Knowledge of when and in which cells each gene is expressed across multicellular organisms is critical in understanding both gene function and regulation of cell type diversity. However, methods for measuring expression typically involve a trade-off between imaging-based methods, which give the precise location of a limited number of genes, and higher throughput methods such as RNA-seq, which include all genes, but are more limited in their resolution to apply to many tissues. We propose an intermediate method, which estimates expression in individual cells, based on high-throughput measurements of expression from multiple overlapping groups of cells. This approach has particular benefits in organisms such as *C*. *elegans* where invariant developmental patterns make it possible to define these overlapping populations of cells at single-cell resolution.

**Result:**

We implement several methods to deconvolve the gene expression in individual cells from population-level data and determine the accuracy of these estimates on simulated data from the *C*. *elegans* embryo.

**Conclusion:**

These simulations suggest that a high-resolution map of expression in the *C*. *elegans* embryo may be possible with expression data from as few as 30 cell populations.

## Background

Multicellular organisms contain many different cell types, each requiring expression of a distinct repertoire of genes. The transcriptome of each cell is regulated by many factors, including signals from neighboring cells [1], long-range gradients of proteins [2], lineage history [3], or environmental conditions. In addition to providing information about cell fate regulation, a gene’s spatial expression pattern may provide clues as to its function. Knowing the timing of gene expression within a cell or lineage provides additional information, such as placing limits on the direction of regulatory relationships between genes. A high-resolution compendium of tissue-specific expression can be used directly to infer regulatory networks, as was done recently for the human hematopoietic lineage [4]. Thus, it would be useful to be able to measure the expression of every gene, in every cell of a multicellular organism, at every developmental time, with different genetic or environmental perturbations.

Existing expression profiling methods have intrinsic tradeoffs; in general, methods that measure expression of more genes have lower spatial or temporal resolution or are less comprehensive in their annotation of distinct tissues. One can measure gene expression with very high spatial resolution in fixed tissues, by staining protein or RNA with affinity reagents. The resulting images can be manually curated to describe where genes are expressed [5]. If the images can be aligned at high resolution, then we get a measure of co-expression in individual tissues, potentially even single cells. This high resolution facilitates analyses such as automated prediction of expression regulation [6]. At the highest spatial resolution, methods such as RNA-FISH allow counting of individual mRNA molecules in fixed tissues [7]. Fluorescent reporters provide a proxy for precisely where and when a given gene is expressed in living cells *in vivo*, and have been used in a wide variety of animal models [6,8,9]. Despite better scalability than affinity probe methods, reporter methods are limited by the rate of transgenesis.

A genome-wide alternative is to isolate tissues or populations of cells from an organism at particular times, and to measure gene expression in each population, using techniques such as microarrays or RNA-seq. This approach has been applied across a wide variety of systems including tissues from human, mouse [10] and *C*. *elegans*[11]. This approach has the advantage of full transcriptome analysis, but spatiotemporal resolution depends on the feasibility of purifying specific cell populations. In addition, the requirement that each tissue or cell population be purified and analyzed separately limits the number of distinct cell types for which expression can be mapped at high resolution across whole organisms.

One strategy to extract high-resolution expression information genome-wide across full organisms or tissues is to integrate data from multiple individual lower-resolution experiments by computational inference. Inference methods take advantage of the fact that genes expressed in a particular tissue or cell population will show expression changes correlated with (possibly subtle) changes in the distribution of cell types in genome-wide expression experiments, even if those experiments aren't designed to be location-specific (e.g. [12]). However, these predictions are limited in resolution by the spatial resolution of the training data, and the amount of inherent spatial information present in available datasets.

Deconvolution methods can be used to determine cell or tissue-specific gene expression patterns from measurements of gene expression in partially overlapping populations of an organism’s cells. One approach is to infer expression in tissues from measurements of mixed tissues, but this typically requires an overdetermined design with at least as many measurements as there are tissues [13]. Others have attempted to use an underdetermined design by combining genome-wide expression measurements from 13 temporal and 14 spatial samples to predict expression in groups of cells in the *Arabadopsis* root [14]. This successfully inferred tissue-specific expression of genes, even in some tissues that hadn't been explicitly measured. This method requires spatial and temporal measurements, such that the spatial measurements are not mutually overlapping (and similarly for the temporal measurements).

### Advantages of deconvolution in the *C*. *elegans* embryo

The nematode worm *C*. *elegans* is an extensively studied model organism with several experimental advantages that make it an ideal animal developmental system for comprehensive gene expression mapping. Each *C*. *elegans* embryo produces 671 cells through an identical pattern of cell divisions, known as an “invariant lineage” [3] and hatches as a L1 larval worm ~14 hours after fertilization. The invariant lineage means that each embryo of a given stage has an essentially identical cellular makeup and that knowing a cell’s lineage history unambiguously predicts that cell’s position in the organism and what tissue identity that cell will adopt. Despite this, the basic body plan, tissue types, and molecular pathways specifying those tissues are frequently conserved with other animals, including humans (e.g. [13,14].) Furthermore, *C*. *elegans* embryonic cells can be dissociated, and cells expressing a fluorescent reporter purified by FACS. The resulting samples can then be analyzed genome-wide for expression by methods such as microarray hybridization or RNA-seq [11,15] and the results related back to the lineage if the identity of the FACS-sorted cells is known.

Many reporter strains are available in *C*. elegans in which cells expressing a particular gene are labeled with a fluorescent protein, allowing visualization of that gene's expression throughout development. We and others have used automated lineage tracing [16,17] to determine the expression of 127 *C*. *elegans* fluorescent reporter strains across each cell in the lineage [9,18]. This lineage tracing approach allowed us to identify all cells expressing each of these reporters. While none of these reporters uniquely identify a single cell, in combination they can distinguish most of the 671 terminal cells in the lineage from each other. This collection of reporters provides a large set of overlapping cell populations that could be analyzed by RNA-seq and used for deconvolution at resolutions approaching single cells. Here, we describe computational methods to infer expression across each cell in the *C*. *elegans* embryo from FACS sorted cell populations, and we test these methods on simulated data to define the accuracy bounds for the expression predictions. Although we focus on estimating gene expression in the developing *C*. *elegans* embryo, the methods are general and may be applicable in other stages of *C*. *elegans* development [8], or in other organisms where reporter overlap can be defined at similarly high resolution, such as *Drosophila*[6].

## Result and discussion

In this study, we test the feasibility of deconvolving expression patterns from genome-wide expression measurements in sorted cells from *C*. *elegans* reporter strains. We propose to sort cells using the collection of reporters for which we previously determined the identity of all expressing cells using lineage analysis. In the remainder of the paper we use the term “fraction” to describe one population of cells that has been purified in this manner and whose constituent cells are known. The overall strategy is then to deconvolve the expression patterns from several fractions to infer the expression patterns at higher resolution, either in individual cells or small groups of cells.

We address a number of questions. How well do different possible methods work for this deconvolution? How accurately can expression be inferred? How many fractions need to be sorted for a given level of accuracy? Can we accurately predict not only the expression levels of a gene across cells, but also the confidence of the predictions? How would experimental noise influence the accuracy of the predictions? We addressed these questions by comparing the performance of several deconvolution methods on synthetic datasets.

### Model

**Figure 1:**
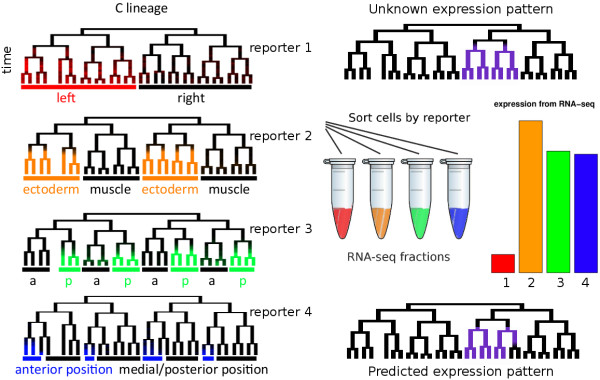
**Illustration of the method.** We assume that we know the expression patterns of a set of reporters (subset of four reporters expression across ~31 terminal cells and their ancestors shown on the left – the full dataset annotates the expression of 127 reporters across all cells). Each expression pattern is drawn superimposed on a lineage tree. These trees show a group of related cells from the *C*. *elegans* lineage with divisions denoted by bifurcations on the on the x axis and time on the y axis. Because of the invariant development, each embryo expressing a given reporter always has reporter expression in the same cells on the lineage, and this is a perfect proxy for cell fate and position. We then flow-sort cells which are expressing each reporter, and perform RNA-seq on the resulting fractions of cells. Based on these measurements, we attempt to estimate expression of each gene in each cell.

Given a reporter expressed in a known pattern, we can sort cells expressing (or not expressing) that reporter and can then measure the total expression of all genes in that fraction (Figure 1). Because each fraction contains a mixture of cells, the measured expression of a gene in a fraction is a linear combination of the expression of that gene in the fraction’s constituent cells.

Suppose there are *n* cells, and the expression of some gene in cell *j* is x_j_ . We wish to estimate x_j_ from measurements of the gene’s expression in sorted fractions from *m* different reporters. Let *A*_*ij*_ be a number between 0 and 1: 0 if sample *i* doesn't contain cell *j*, and 1 if it does; we refer to this as the *sort matrix*. Let *b*_*i*_ be the total expression of a gene in fraction *i* . Then we can cast this as an (underdetermined) constrained linear regression problem:

Ax=b,wherex≥0

Given that the expression values also were constrained to be positive, the possible expression values form a convex region in a linear space; the size of this space represents confidence in the expression levels in each cell. For example, the reporters shown in Figure 1 correspond to the system of linear equations:

$$\left[\begin{array}{llllllllllllllll} 1 & 1 & 1 & 1 & 1 & 1 & 1 & 1 & 1 & 1 & 1 & 1 & 1 & 1 & 1 & 1\\ 1 & 1 & 1 & 1 & 1 & 1 & 1 & 1 & 0 & 0 & 0 & 0 & 0 & 0 & 0 & 0\\ 1 & 1 & 1 & 1 & 0 & 0 & 0 & 0 & 1 & 1 & 1 & 1 & 0 & 0 & 0 & 0\\ 0 & 0 & 1 & 1 & 0 & 0 & 1 & 1 & 0 & 0 & 1 & 1 & 0 & 0 & 1 & 1\\ 1 & 0 & 0 & 0 & 1 & 0 & 0 & 0 & 1 & 0 & 0 & 0 & 1 & 0 & 0 & 0 \end{array}\right]x=\left[\begin{array}{c} \mathrm{all}\\ \mathrm{fraction}1\\ \mathrm{fraction}2\\ \mathrm{fraction}3\\ \mathrm{fraction}4 \end{array}\right],\mathrm{where}\;x\geq 0$$

Depending on the available reporters and the expression pattern of the gene under consideration, such data may indicate the exact expression pattern. For example, if a gene is expressed in only one of the 1,341 embryonic cells, an ideal set of measurements in log_2_(1,341) <11 sorted fractions would be enough to distinguish which is the expressing cell, as each fraction could potentially “rule out” expression in half of the cells. While expression in a single cell does occur (e.g. [19]), most genes are expressed in broad collections of cells rather than individual cells, and in practice, the reporters available for sorting do not match this ideal set.

### Simulations

We tested the performance of different deconvolution algorithms on several synthetic expression datasets. Each dataset contained from 123 to 371 synthetic genes for which the true expression across all embryonic cells was known. We then generated simulated expression measurments for each of these genes in each fraction, by summing expression in the fractions containing the cells positive or negative for reporters whose expression pattern across all cells we determined previously [9].

We wanted to test whether methods could correctly deconvolve expression of patterns similar to those seen previously, as well as novel patterns. We expect the accuracy of a method for deconvolution to depend on the expression pattern being predicted, with simple patterns or patterns similar to the sort markers being easier to predict. We therefore measured accuracy on an expression dataset including 123 of the known reporter expression patterns [9], augmented with several synthetic patterns (Additional file 1: Figure S1). One collection was designed to have a random expression pattern, such that the overall correlation between cells was similar to the correlation structure of the known expression patterns. For example, in real expression patterns, cells with very close lineal relationships, similar tissue identities, or left-right symmetric equivalents are more correlated in their expression than random cells. We also generated a collection containing each pattern corresponding to expression in a single cell or lineage. Finally, because most *C*. *elegans* cells exist as left-right symmetric pairs [3], we also generated patterns with expression in each left-right lineage pair. While we cannot simulate every possible expression pattern, these data sets should be representative of the diversity of expression patterns that may exist.

### Choice of fractions

The performance of a deconvolution method likely depends on both the total number of fractions assayed, and which fractions are analyzed. While accuracy may be highest if all 127 fractions were analyzed, assaying that many fractions would be expensive and time-consuming. Ideally, we would like to identify collections of fractions that maximize the accuracy of deconvolution. Compressive sensing theory suggests that any orthogonal set of expression patterns should perform well [20]. To select such a set, we designed a greedy approach to iteratively choose fractions to analyze from the reporters with known expression patterns [9]. We chose reporters based on which maximizes the accuracy of predictions, as defined by correlation coefficient, on the collection of 371 patterns with expression in one lineage. A single set was selected using the simplest deconvolution algorithm, the naïve pseudoinverse (see below). The reporters chosen for sorting by this method tended to be orthogonal; of the first 30 reporters chosen, the mean absolute correlation between pairs was 0.15 (very similar to 0.17, for all pairs of reporters). Reporters chosen by this method were slightly more accurate than randomly chosen reporters (data not shown). We used this same ordered list of reporters in evaluating all of the deconvolution methods on all of the simulated datasets.

### Methods for deconvolution

We tested deconvolution methods based on two general approaches: the pseudoinverse and expectation propagation (EP). We describe each strategy and their variations below, then overview the performance of the different methods on the simulated data.

#### *The pseudoinverse*

In our simulations, the expression of each gene in each fraction is described by a potentially underdetermined linear system of equations, as there are more cells than available fractions. The Moore-Penrose pseudoinverse provides a single solution to such a system based on a minimal least-squares fit. However the solution obtained by calculating the pseudoinverse may contain negative entries, corresponding to the biologically unmeaningful “negative expression.” We thus tested two variants of the pseudoinverse that produce only positive solutions. We either replaced negative numbers with zero, referred to as the “naïve pseudoinverse,” or incorporated the constraint that expression is positive along with the linear constraint, referred to as the “constrained pseudoinverse.”

Compressed sensing theory states that it can be possible to reconstruct a signal from fewer measurements if there is some regularity to that signal [20]. In existing data, cells sharing similar lineage histories, symmetry relationships or tissue types are more likely to have similar gene expression [9]. To take advantage of this, we tested an additional variant of the pseudoinverse which weights potential solutions based on the covariance between each pair of cells, as estimated from the known gene expression patterns.

#### *Expectation Propagation*

We also deconvolved expression by using Expectation Propagation (or “EP”), which is an iterative strategy for approximating a probability distribution [21]. Unlike the pseudoinverse, EP predicts a range of possible expression patterns compatible with the data, and thus provides an intrinsic estimation of the confidence of the prediction. When comparing accuracy between EP and pseudoinverse-based methods, we used the mean of the EP solution. Although the iterative steps in EP usually converge, they sometimes diverge, resulting in numerical problems, and no prediction. For instance, predictions for 10 of 127 genes failed to converge when predicting the real expression patterns with 75 fractions, and 27 genes failed to converge when predicting with 100 fractions (Table 1). In general, EP's convergence is difficult to prove; failure to converge may indicate that the approximating distribution doesn't fit the posterior well [22]. Many of the cases in which convergence failed were cases in which only a few cells were expressing; suggesting that these cases may be poorly fit by the approximating distribution. We found that we could increase the convergence rate by adding a damping step, and modifying the algorithm to report the expression predictions of the last iteration irrespective of convergence. This produced an answer in all cases, but resulted in slightly lower accuracy (about 5% lower correlation on the actual expression patterns with 30 reporters), and was about eight times slower, compared to the undamped version. Computing the EP prediction required more CPU time than the naïve pseudoinverse, but was faster than the other methods when accounting for the time required to estimate the confidence of deconvolution (Table 2).

**Table 1:** Number of problem instances in which EP failed to converge

**Dataset**	**Number of fractions**	**Number of cases which failed to converge**
measured expression (n=123 synthetic genes)	10	2 (2%)
"	75	10 (8%)
"	100	27 (22%)
synthetic patterns based on correlation (n=200 synthetic genes)	50	2 (1%)
"	75	8 (4%)
"	100	49 (25%)
synthetic one-lineage patterns (n=371 synthetic genes)	100	1 (0.3%)
synthetic two-symmetric-lineage patterns (n=245 synthetic genes)	100	2 (0.8%)

(EP converged in all other cases).

**Table 2:** **Comparison of running time per gene for various deconvolution methods** (**on a machine with a 2**.**4 GHz Intel Xeon processor**, **and 4 GB RAM**)

**Method**	**time (seconds)**
naïve pseudoinverse	0.01
EP	0.5
constrained pseudoinverse	19
constrained pseudoinverse with correlation	23
sampling	583

### Accuracy of deconvolution increases with number of fractions

**Figure 2:**
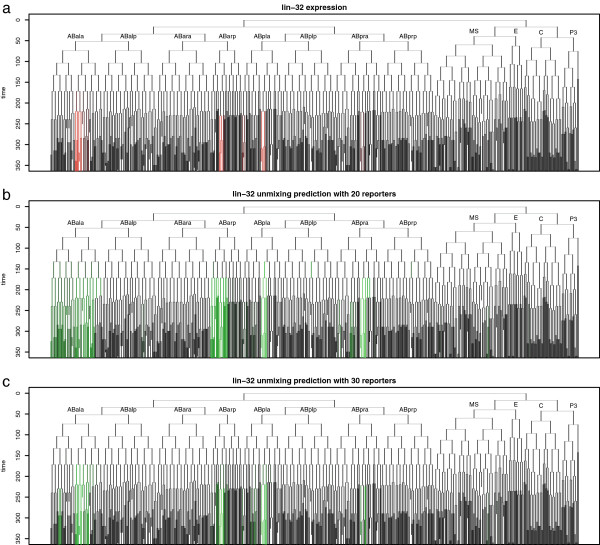
**Example of expression prediction.** Predictions are displayed as lineage tree (ancestry relationships of all cells), using the naming conventions of Sulston [3]. **a)** Measured expression of *lin*-*32* (red) [9]. **b)** Predicted expression using twenty reporters (green). **c)** Predicted expression using thirty reporters (green).

We measured the accuracy of each algorithm's predictions both in quantitative terms, and as classification accuracy of on-off predictions. For each of the simulated data sets, we simulated the measurements from each FACS-sorted fraction. We then applied each deconvolution algorithm, and compared the simulated expression patterns with the predicted pattern from deconvolution. When deconvolving expression for a gene in the known expression pattern set, we excluded that gene from also being used as a sort marker for a fraction, replacing it with the next fraction on the list if necessary. We observed that in many cases, the deconvolved pattern was visually similar to the true pattern, and that the precision of the prediction increased with the number of fractions. For example, Figure 2 shows a measured expression pattern (for the gene *lin*-*32*), and expression predicted by the constrained pseudoinverse method, using either 20 or 30 fractions.

**Figure 3:**
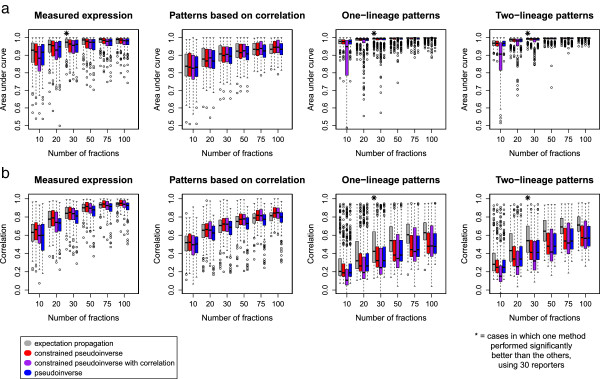
**Prediction accuracy for several real and simulated data sets**, **measured by a) area under the ROC curve or b) Pearson correlation.** An area under the curve of 0.5 corresponds to random on-off predictions, while an area under the curve of 1 corresponds to perfect prediction accuracy. (Data sets are described in the text). Cases in which one method performed significantly better than the other three (paired *t*-test, *p*=0.05, Bonferroni-corrected for 24 tests) are marked with a star.

We first assessed which methods most accurately determine which cells are on or off, without regard for level. We made binary predictions by thresholding the quantitative predictions, and compared these by using the area under the receiver-operating-characteristic (ROC) curve (Area Under Curve (AUC); Figure 3a). This measures the sensitivity-specificity tradeoff for different thresholds of the predictions. An AUC of 1.0 indicates that all expressing cells are predicted to have higher predictions than all non-expressing cells, while an AUC of 0.5 would be expected from completely random predictions. By this metric, EP performed slightly better than all of the other methods on each simulated dataset.

To quantify this similarity of expression levels between real and deconvolved patterns, we calculated the Pearson correlation between the original pattern and the deconvolved prediction (Figure 3b). By this measure, the constrained pseudoinverse gave the highest accuracy on the “measured expression” and “simulated patterns based on correlation” datasets, although the differences with EP were not statistically significant. In contrast, the mean of the EP prediction performed significantly better on the simulated one- and two-lineage datasets. In these experiments, adding the covariance constraint to the pseudoinverse predictions didn't improve accuracy; instead it reduced accuracy for one- and two-lineage patterns, possibly because these patterns are fairly different from the patterns used to compute the correlation matrix. The constrained pseudoinverse (with or without the correlation-based prior) performed best when predicting the random patterns generated from the correlation distribution calculated for real genes.

The one- and two-lineage datasets were simulated with a low level of normally-distributed noise. To test accuracy with non-normal distributions, we repeated the EP simulations, with “on” and “off” levels randomly drawn from gamma distributions (Additional file 2: Figure S2). The results from this with lower levels of noise were comparable to results using normally-distributed noise, although higher levels of noise decreased accuracy considerably.

For all methods, adding additional fractions increased accuracy by either AUC or correlation. Eventually, the accuracy began to plateau with very little improvement with more than 50 fractions, and the biggest improvements in accuracy at less than 30 fractions. We conclude that for most patterns, EP deconvolution appears to be a slightly more accurate approach, and that while more fractions is better, at least 30 fractions are needed to approach the rate of diminishing returns for deconvolution across the entire lineage.

### Confidence measurements accurately predict error bounds for predictions

An ideal deconvolution method would include some estimate of the confidence of its predicted patterns, because some patterns are likely to be predicted with higher confidence than others. For the pseudoinverse-based methods, we used a sampling approach to estimate confidence, while EP gives a direct measure of uncertainty. We tested these methods for measuring confidence and compared the predicted confidence to the measured deconvolution error across the simulated datasets.

**Figure 4:**
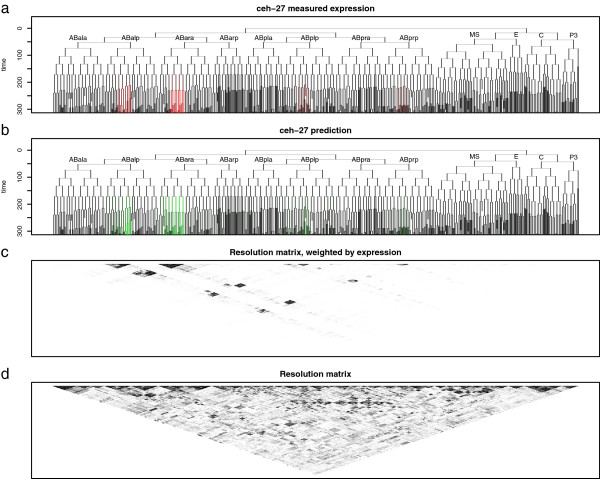
**Expression prediction for *ceh-27 *computed using expectation propagation (EP), showing a) the actual expression pattern (red), b) the predicted expression pattern (green), c) the resolution matrix weighted by expression, and d) the resolution matrix.** Dark blocks in the expression-weighted resolution matrix indicate potentially conflated expression predictions.

The process of combining expression from groups of cells, and then deconvolving using the naïve pseudoinverse, is a linear transformation. This transformation can be represented as a matrix (*A*^†^*A*, where *A*^†^ is the pseudoinverse of the sort matrix, *A*), known in geophysical modeling as the model resolution matrix [23]. This resolution matrix depends on both the sort markers used, and the underlying expression pattern for a given gene, resulting in a distinct resolution matrix for each deconvolved gene. As we add linearly independent reporters, the resolution matrix approaches the identity matrix. Large blocks on (or off) of the diagonal represent sets of cells which the experimental design has difficulty distinguishing and for which expression is “blurred” together (Figure 4). This provides a graphical display of which cells’ expression values are conflated for any given gene.

**Figure 5:**
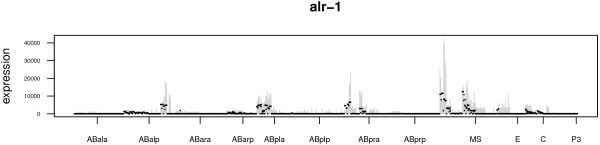
**Prediction bounds for a typical gene, *alr-1, *computed using the Constrained Pseudoinverse and Markov Chain Monte Carlo Sampling, based on simulated measurements of thirty fractions.** Actual expression is shown in black, while grey bars show predicted expression (as a two-standard-deviation interval).

**Figure 6:**
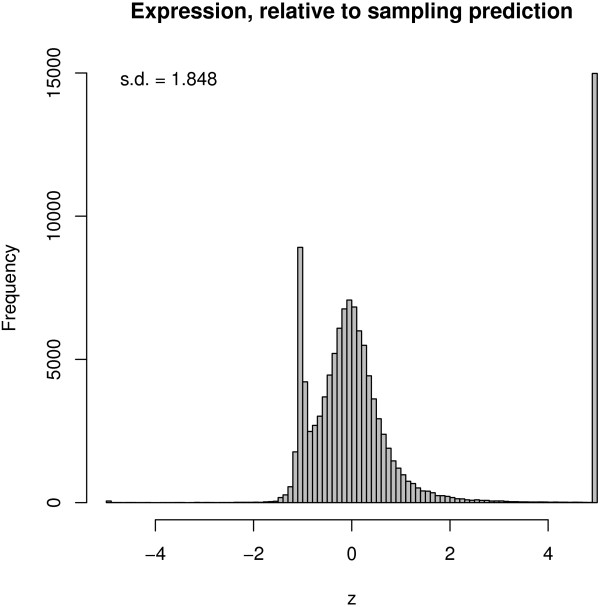
**Comparison of sampling prediction intervals with actual expression.** z-score of actual expression was plotted, scaled to the mean and standard deviation of the prediction from sampling. For example, if the real error were equal to the prediction interval standard deviation, then the z-score would be 1. Values outside of ± 5 are shown at ± 5.

The uncertainty of the pseudoinverse predictions can be predicted by sampling. When using the pseudoinverse with the constraint that expression is positive, the possible solutions form a convex region in a linear space. While the true solution could be anywhere in this region, one model of prediction uncertainty is to assume uniform probability across the region. We used Monte Carlo Markov Chain sampling [24] to approximate the range of possible expression patterns. Specifically, we used random-directions sampling, which is guaranteed to mix eventually when sampling from a convex region, although the amount of sampling needed depends on the shape of the region [25]. These error bounds usually, encompassed the true expression pattern (Figure 5). However, this was computationally demanding enough that it would be slow (but not impossible) to apply genome-wide (Table 1). Sampling also occasionally underestimated the uncertainty by not including the entire feasible solution space (Figure 6) (10% of estimates had z > 4).

**Figure 7:**
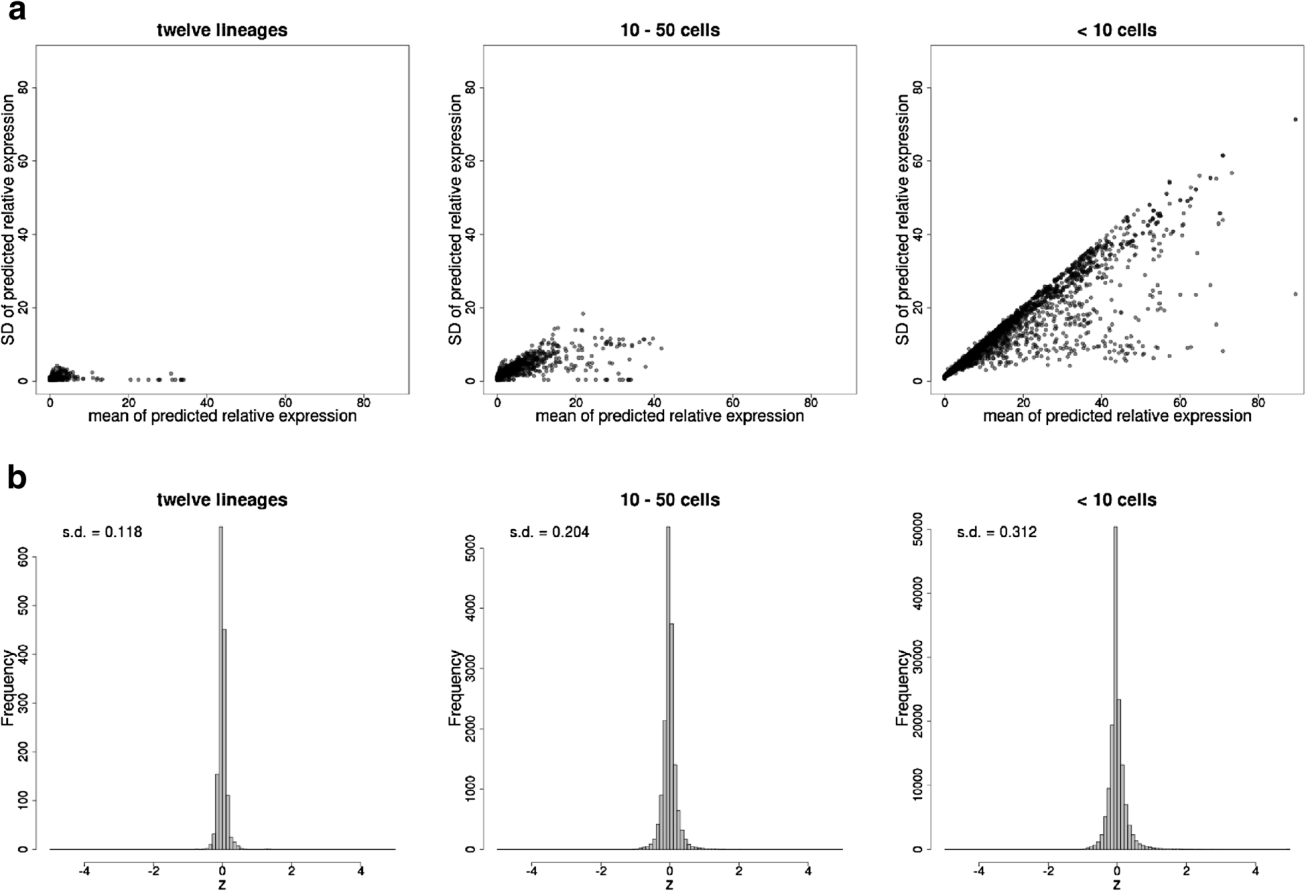
**Sizes of EP prediction bounds for 123 genes, using thirty simulated reporters. a)** Mean and standard deviation of predictions for three different sizes of groups of cells. Larger groups of cells correspond to lineages with many cells (prediction is for whether expression is in that lineage, but not which cell within the lineage). All possible lineages were analyzed for each gene. **b)** Actual expression, scaled to the mean and standard deviation of the prediction to produce z scores as in Figure 6. Values beyond ± 5 are shown at ± 5.

In contrast to the pseudoinverse, the EP approach provides an intrinsic measure of uncertainty because it predicts expression to occupy a convex region, which is approximated by a multivariate normal distribution in a linear space [21]. The marginals of this distribution provide a potential estimate for the uncertainty of each cell’s expression prediction. We plotted the mean and standard deviation of the expression predictions for each gene in each cell (Additional file 3: Figure S3b). Few cells have error bounds which were confidently greater than zero, probably because we sometimes cannot distinguish low expression in a group of cells from high expression in a few of them. However, we reasoned we might be able to make more confident predictions for groups of related cells. To test this, we estimated the total expression in lineage groups of cells, by summing part of the mean and covariance obtained by EP across sublineages. For instance, we can estimate the mean expression of a gene, in all cells in a particular lineage (Additional file 3: Figure S3c). In most cases, this allowed the identification of specific lineages where there was high confidence of expression somewhere in that lineage. Such predictions of total expression in larger groups of cells are narrower, as they don't attempt to predict precisely which cells express a given gene (Figure 7a).

We modeled the deconvolution error by normalizing each expression measurement by the prediction standard deviation. The resulting distribution resembles a normal distribution with a mean of zero and standard deviation less than 0.31 both for small and large cell groups (Figure 7b). This suggests that EP is conservatively estimating the confidence of its expression predictions.

We also compared the uncertainty estimates computed using the sampling to those computed by EP. The regions computed using sampling had comparable means, but smaller standard deviations by a factor of about 2 (Additional file 4: Figure S4). Comparing the uncertainty estimates with the actual error in the predictions indicates that the sampling uncertainty estimates are narrower than the range of possible solutions, and that the EP uncertainty estimates are wider than the actual possible region. EP provides a prediction based on a multivariate normal distribution, while real expression levels are likely not to be normally distributed. Nonetheless, we found that the mean and standard deviation of the EP uncertainty bounds were highly correlated (Pearson *r* of 0.96 and 0.93, respectively) with those produced by sampling. This suggests that these metrics are not strongly affected by this assumption. We conclude that in addition to providing more accurate deconvolution for most patterns as described above, the EP method also provides accurate, and possibly more conservative, uncertainty estimates compared with sampling, and is computationally more scalable than sampling-based approaches.

### Prediction accuracy is sensitive to sort-matrix errors but robust to measurement noise

The simulations described so far have assumed that the gene expression levels themselves have noise but that we have noise-free information about which cells are present in each fraction and about expression levels in each fraction. In practice, some level of experimental error in these measurements is unavoidable. Therefore, we assessed the methods' ability to tolerate various kinds of noise by perturbing different parts of the input data and measuring the resulting effect on prediction accuracy. All of the noise simulations were performed using a set of 30 sort fractions.

**Figure 8:**
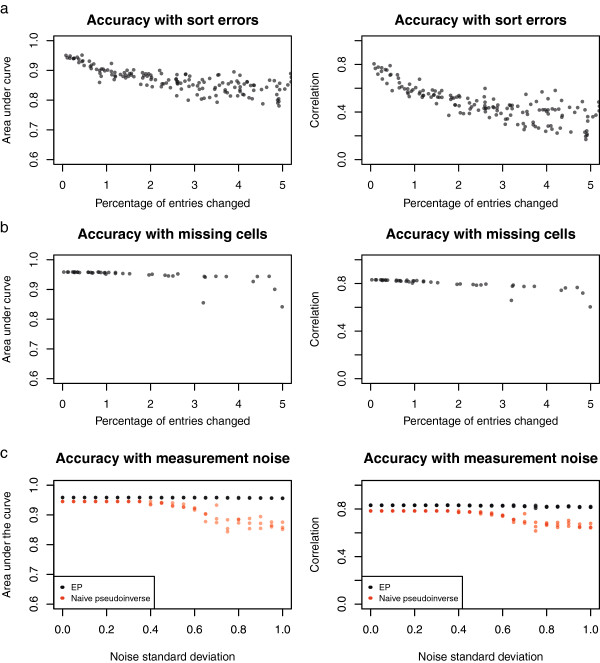
**Deconvolution accuracy, by AUC and correlation, in the presence of various kinds of experimental noise. a)** Accuracy when some elements of the sort matrix are incorrect. **b)** Accuracy with the sort matrix perturbed by removing some cells from all measurements. (In **a)** and **b)**, the *x*-axis represents the total number of entries in the sort matrix which were perturbed.) **c)** Accuracy when deconvolving with measurements perturbed by random noise (three different averages for each noise level are shown).

It is possible that errors in the lineage data or experimental differences between FACS and confocal microscopy could introduce errors into this step. Therefore we tested how sensitive the deconvolution approaches are to errors in the sorting assignments by randomly perturbing different entries in the sort matrix, without making compensatory changes to the simulated expression data. This treatment mimics the situation when some cells are systematically sorted into a different fraction than predicted. Even minor perturbations of the sort matrix reduce accuracy, whether measured by correlation or area-under-the-curve (Figure 8a), with a roughly 3% decrease in AUC accuracy (or 16% decrease in correlation accuracy) for each 1% increase in systematic sort error. Thus, in any application of this deconvolution approach, it will be important to accurately determine the sort matrix. In contrast to this systematic sort error, deconvolution is robust to random noise in sorting, especially if the amount of random sort error is known (as can be measured directly by resorting FACS-sorted cells) and included in the sort matrix used for deconvolution (data not shown).

It is also possible that specific cells or cell types could be lost during the dissociation and FACS sorting process. For instance, large cells present in the early embryo might be removed by filtering steps, or may be damaged by shear forces during the isolation of single cells [26]. If FACS approaches to remove cell clumps by gating on forward and side-scattered light are employed, these approaches may also eliminate real cells with complex morphologies. To estimate the effects of this type of error, we simulated a sort process where some cells were specifically lost, and then deconvolved the resulting perturbed measurements without knowledge of which cells were lost. The EP method was fairly robust against such errors (Figure 8b), even when up to ~25% of cells (300) were missing.

Measurements of expression include both biological variability, such as differences in growing conditions between embryos, and technical variability, such as variation in RNA amplification, sequencing biases and random noise resulting from sampling of sequence reads. To estimate the effects of measurement noise, we simulated deconvolution with each fraction's measurement in the simulated expression dataset scaled by various levels of random noise (Figure 8c). The EP method was very robust against such noise, with little decrease in either quantitative accuracy or classification accuracy even with a noise standard deviation of ~1 (corresponding to roughly 2-fold average error in the expression measurements.) The naïve pseudoinverse was somewhat more sensitive to such noise.

In conclusion, we find that the EP algorithm gives the most reliable deconvolution of expression values in single cells from mixed cell populations, and provides accurate uncertainty estimates in a computationally tractable manner. Systematic loss of particular cell types or random measurement noise have little effect on overall deconvolution accuracy. However, errors in the assignment of cells to sort fractions do decrease accuracy, suggesting that optimizing this parameter is critical in experimental application of these methods.

## Conclusion

We have described a method for deconvolving gene expression in a large number of single cells, starting from a smaller number of measurements in overlapping fractions of cells. Our simulations indicate that for *C*. *elegans* embryos, the fact that we have many orthogonal reporters for use as sort markers should make it possible to deconvolve expression with good accuracy from a fairly modest number of sort fractions. The same strategy is also applicable to other sorts of measurements for which a global collection of measurements across cells would be useful, such as ChIP-seq and proteomic assays. All methods based on cell-sorting are subject to the caveat that FACS sorting can cause cell death, and alter measurements of properties such as gene expression, so observed expression patterns should be confirmed *in vivo*. Similar deconvolution should be possible in other systems where the overlap of different markers can be determined with high accuracy, such as in the *Drosophila* blastoderm [6].

Our predictions are not exact, but do provide an estimate of their uncertainty. Surprisingly, the deconvolution is fairly robust to certain types of measurement noise, such as random noise in the expression measurements and loss of specific cells during sorting into fractions. Not surprisingly, the method is more sensitive to systematic errors in the sort matrix that indicates which cells are present in which fraction. Together this suggests that while deconvolution may be possible with fairly modest numbers of replicates for each sort fraction, the cells present in each fraction must be well-defined. This can be accomplished by only using fractions based on fluorescent reporters that show clear on-off patterns of expression (as opposed to quantitative patterns that may be harder to gate for sorting).

The accuracy and efficiency of deconvolution could be further improved by focusing on a smaller subset of cells in the organism. The *C*. *elegans* embryonic cells can be divided into 12 sublineages of ~100 cells based on their descent from a common founder cell. Simulation data suggests that expression patterns in these sublineages could be deconvolved with similar accuracy to that reported here with even fewer (~10-15) reporters (data not shown). Additional improvements could be obtained by the availability of more sort markers, either by using lineage tracing to annotate the expression of more reporters, or by using existing different color (e.g. GFP and RFP) reporters for multicolor sorting to collect smaller fractions of cells based on coexpression of two or more markers.

The EP method provided predictions with competitive accuracy, including an estimate of confidence, at moderate computational cost. One challenge of EP is that it doesn’t converge in all circumstances. In our simulations, EP generally converged in circumstances with fewer than fifty reporters, which are sufficient to give reasonable accuracy across the entire lineage. In cases in which EP doesn't converge, we modified the method to use damping or to show the non-converged prediction. The sampling method also appeared to give reasonable estimates of confidence. Applying the current sampling method genome-wide would require 1,600 CPU hours (assuming 10,000 *C*. *elegans* genes are tissue-specific), which is expensive but not prohibitively so, even without using methods such as adaptive sampling [24] to accelerate it.

Several related studies (reviewed in [27]) attempt to deconvolve expression measurements from mixed tissues. Most of these assume, like us, that measurements are linear combinations of tissues [28]. One related method is [29], which combines a set of non-overlapping spatial measurements with a set of non-overlapping temporal measurements, and assumes these are independent, resulting in an overdetermined problem. However, our model differs by allowing measurements that may or may not be independent, and by treating the problem as underdetermined. Our current model can also incorporate explicit temporal data by including sort matrix entries corresponding to cells at a particular time. Its temporal resolution could be improved by integrating existing embryonic time course data [30], using methods specifically designed for timeseries data [31,32].

Another class of existing deconvolution methods infer the components of a mixture based solely on expression profiles [33,34]. These approaches don't require purification of cells but may not be applicable to the overlapping fractions in our setting or to organisms like *C*. *elegans* where the cellular composition of intact tissues is invariant between samples from the same developmental stage. Furthermore, they don't allow explicit incorporation of the information about mixture compositions we obtained from imaging data. Other methods estimate the proportions of a mixture, assuming expression profiles of its components are similar to known reference expression profiles [27,35]; in our case, such reference expression profiles aren't available.

Alternative approaches become available if we can measure expression in many more cell populations than there are cells (in this case, >~1,341 measurements). For example, csSAM [36] and DSection [37] estimate expression in groups of cells from measurements of mixtures of cells with unknown (or partially known) proportions using regression. However, this method requires many more samples than are feasible with current methods in *C*. *elegans*. The methods used in that model might be adapted to our situation, especially if methods are developed to allow expression profiling of extremely large numbers of cell populations. With the methods we describe and the increasing availability and decreasing cost of sequencing, a comprehensive description of expression patterns across all cells of a developing organism may soon be possible.

## Methods

### Sort matrix

We based our sort matrix on per-cell expression intensities of fluorescent reporters [9]. We classified cells as “on” or “off” using a logistic model, in which “off” cells had intensity with mean 0 and standard deviation 1,000, and “on” cells had intensity with mean 2,000 and standard deviation 1,000. In some cases, this resulted in probabilistic sort matrix entries between 0 and 1 (which is compatible with all the methods we tested).

### Synthetic datasets

We measured accuracy using expression data with cellular resolution from 123 of the 127 fluorescent reporters in [9]. We also measured accuracy on three synthetic data sets (Additional file 1: Figure S1):

•Synthetic expression data, drawn from a multivariate normal distribution with mean 0, and covariance estimated from the expression of those reporters.

•Synthetic expression, in which one lineage of cells is “on” (with expression randomly drawn from a normal distribution with mean 0 and variance 1), and the others are “off” (with expression randomly drawn from a normal distribution with mean 10 and variance 11.) There are 371 such lineages containing at least five cells.

•Synthetic expression in which two symmetric lineages are “on” or “off”, as above. There are 245 such lineage pairs in which each lineage contains at least five cells.

In all cases, negative expression values were truncated to zero.

### Naïve pseudoinverse

Our simplest prediction was *A*^+^*b* , where *A*^+^ is the Moore-Penrose pseudoinverse of *A*. This prediction is the solution to *Ax*=*b* having minimum 2-norm. We truncated negative entries of this solution at zero (although doing so will, in general, violate the linear constraint).

### Constrained pseudoinverse

We can also incorporate the constraint that *x*≥*0* while solving for *x*, finding the maximum likelihood estimate of n

x∼N(0,I)subject toAx=b,x≥0

(Since the covariance is *I* , this is equivalent to finding a value of x which satisfies the constraints, and minimizes the 2-norm of x .) We used the lsei R function to solve this problem as this includes explicit equality contraints. We also tested an alternative R function, nnls. This is more complex because it requires encoding the constraints in a cost function, but has the advantage of being around ten times faster, and gave similar results.

### Pseudoinverse deconvolution with correlation constraint

To include correlation in our model, we assumed that x has a normal distribution with known covariance Σ :

x∼N(0,∑)subject toAx=b,x≥0

We estimated correlation based on 123 of the known reporter expression patterns. We used a shrunken estimate of correlation, from the corpcor R package [38], and manually set the shrinkage value to 0.05 (the default shrinkage value estimated by the corpcor package resulted in a very flat correlation.) Again, we used the lsei R function to estimate the most likely value for x.

### Sampling

We used random-direction Markov chain Monte Carlo sampling. Initially we used the xsample function (with the “cda” option) from the limSolve package [39]; we then re-implemented the core of the algorithm in C++ using the Rcpp package [40]. We used the mean and variance of ten million iterations as our prediction, after ten million iterations of burn-in. (We computed statistics on chains thinned to every 1,000^th^ sample.) We omitted cells from sampling which had zero expression according to the constrained pseudoinverse method; without this restriction, sampling failed (as the distance it could move in the random direction was zero.) Chains from multiple starting points appeared to have converged after 50 million samples, by eye (Additional file 5: Figure S5), and the potential scale reduction *R* was typically less than 1.1 (Additional file 6: Figure S6), suggesting convergence ([24], pp. 296–298).

### Expectation propagation

We approximated the possible range of expression using Expectation Propagation (or “EP”), which is an iterative strategy for approximating a probability distribution [21]. In our case, we approximated the region of possible expression with a multivariate normal distribution. We used a parallel updating strategy, repeatedly updating our estimate of each cell's expression so that x≥0, then altering our estimate to satisfy the constraint that *Ax*=*b*[41]. (Our implementation of this, and the other deconvolution methods, is available as Additional file 7).

Convergence of EP is known to be problematic, especially when the approximating distribution is a different shape from the posterior [21]. On smaller synthetic problems, the mean and standard deviation of the regions estimated by the method agreed well with the distributions estimated by the xsample function [39] (data not shown.) However, when estimating 1,341 numbers, the algorithm sometimes failed to converge. We addressed this by incorporating a prior with variance 100 times the total expression. We also added 10^-3^ to each cell's relative expression (and subtracted this off from the solution afterwards.) With these modifications, EP often, but not always, converged (Table 1).

We also experimented with a damped version of EP, by adding a step size, initially 1. At each step, we scaled the EP update by this amount. If an update would lead to numerical errors, we divided the step size in half, and continued from the last estimate.

### Error simulations

For simulations of error, we measured the EP method's accuracy on 123 known expression patterns, using thirty reporters. To simulate errors in the sort matrix, we randomly chose lineages in individual fractions, and replaced each entry α in those lineages with 1-α. To simulate missing cells, we again chose random lineages, and replaced each entry in those lineages (in all fractions) with 0. We then computed expression with this perturbed matrix, and measured accuracy given these perturbed expression measurements (but the original sort matrix.) To simulate noise in expression measurement at a level *s*, we multiplied each expression measurement by random draw from a normal distribution with mean 1 and standard deviation *s*.

## Abbreviations

EP: Expectation propagation.

## Competing interests

The authors declare that they have no competing interests.

## Authors' contributions

JM conceived the study, JM and JB developed the methods and JB implemented them, JB and JM wrote the manuscript. Both authors read and approved the final manuscript.

## Authors' information

JB is a graduate student in the Genomics and Computational Biology program at the University of Pennsylvania.

## Supplementary Material

Additional file 1: Figure S1 Examples of synthetic expression patterns used to measure accuracy. a) Patterns based on correlation. b) Patterns with one lineage on. c) Patterns with two symmetric lineages on. Click here for file

Additional file 2: Figure S2 EP accuracy for one- and two-lineage patterns, measured using a) AUC or b) correlation. Thirty sorted fractions were used. The “off” distribution was drawn from a Gamma(1,1) distribution, and the “on” distribution was the gamma distribution with shape and scale shown on the *x*-axis. Click here for file

Additional file 3: Figure S3 Prediction bounds for expression of a gene in groups of cells, computed using expectation propagation. Thirty simulated reporters were used. a) Measured expression of *unc*-*130*. b) Mean (red) and standard deviation (green) for expression prediction (yellow indicates a large mean and standard deviation.) c) Mean (red) and standard deviation (green) for the average expression in the lineage rooted at a given cell. Click here for file

Additional file 4: Figure S4 Comparison of **a**) mean and **b**) standard deviation of prediction bounds from sampling and EP, for 123 genes, using thirty simulated reporters. Click here for file

Additional file 5: Figure S5 Two-standard deviation posterior predicted intervals for *alr*-*1*, based on mean and variance of increasingly long sampling chains. (Negative values for bounds are truncated at zero). Click here for file

Additional file 6: Figure S6 Potential scale reduction *R* ([26], pp. 296–298) for *alr*-*1*, using increasingly long sampling chains. (Cells whose expression was predicted to be zero by the truncated pseudoinverse method were not included in the sampling, and are not shown). Click here for file

Additional file 7 R source code implementing the deconvolution methods (as a .zip archive). Click here for file
