# Supplementary figures and images for: Deconvolution of gene expression from cell populations across the C. elegans lineage
Source: BMC Bioinformatics. 2013 Jun 22;14:204. doi: 10.1186/1471-2105-14-204 (PMC3704917; doi:10.1186/1471-2105-14-204)

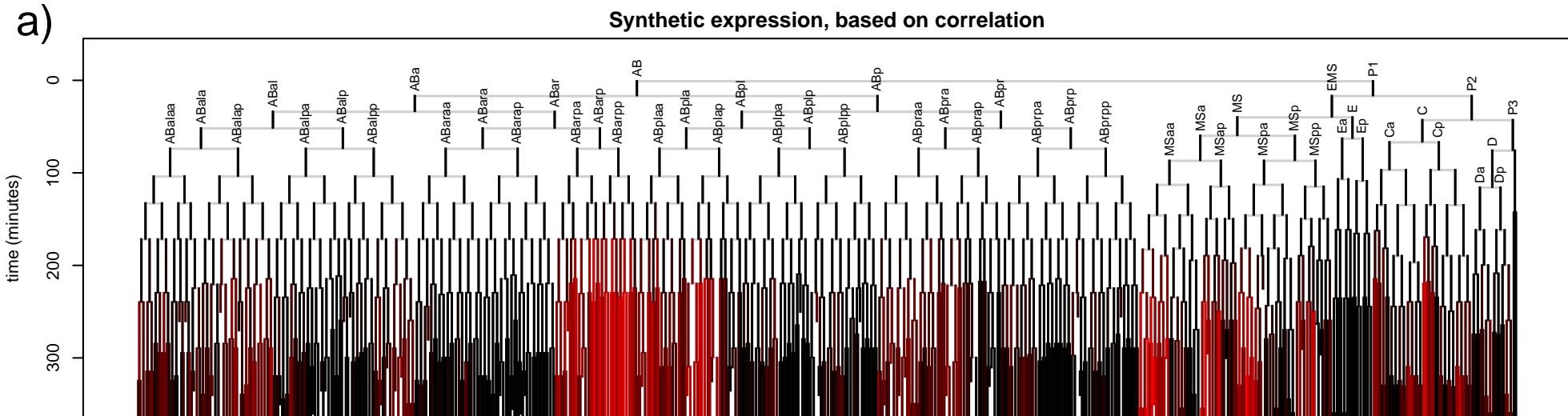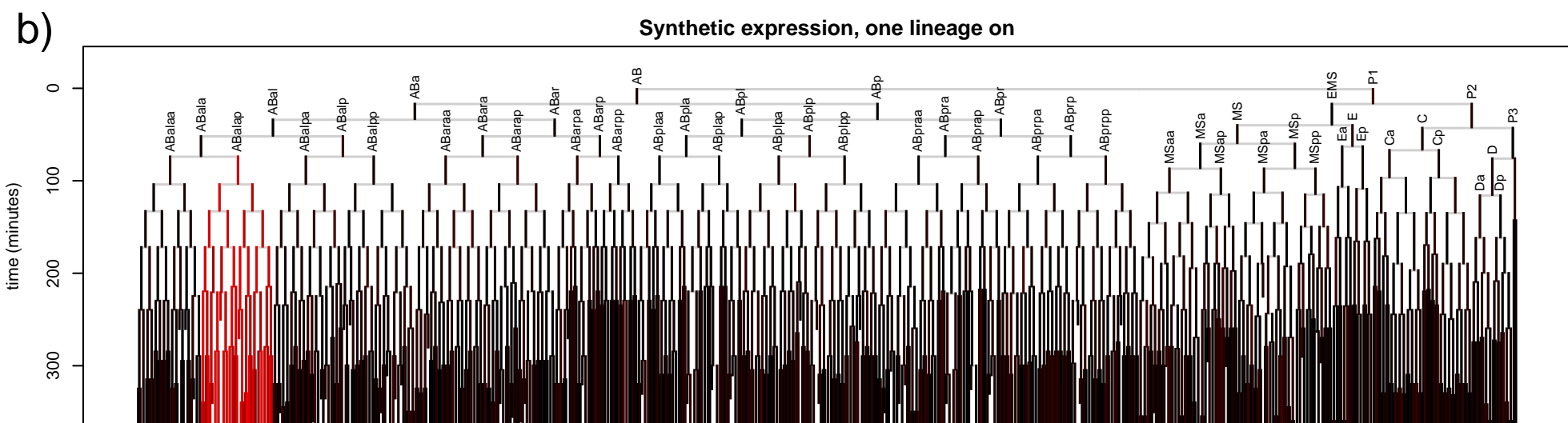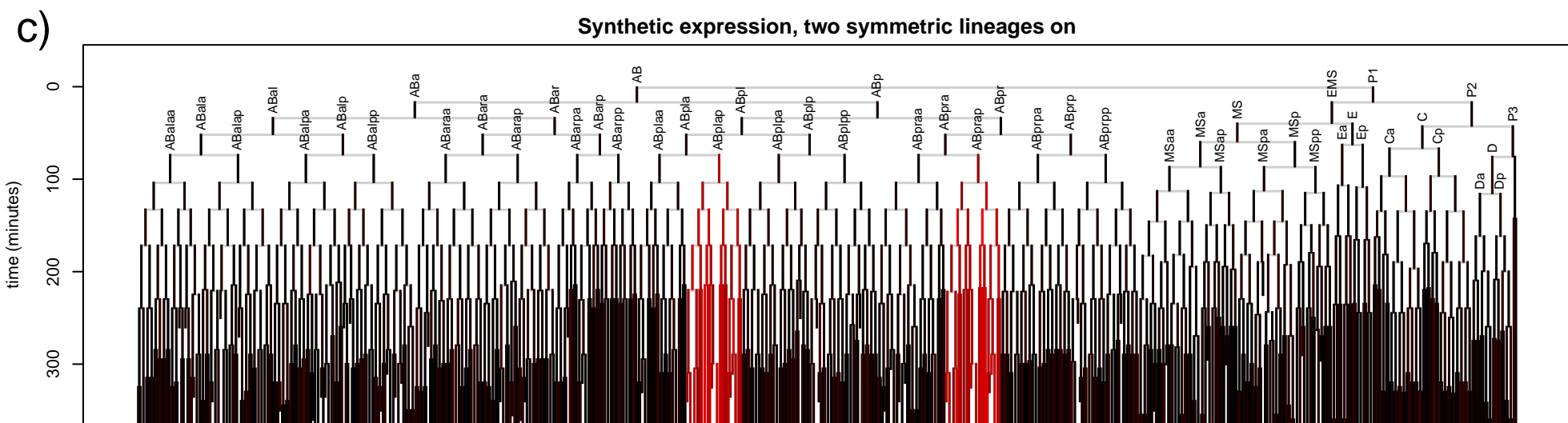

Supplement: Additional file 1: Figure S1 — Examples of synthetic expression patterns used to measure accuracy. a) Patterns based on correlation. b) Patterns with one lineage on. c) Patterns with two symmetric lineages on. [file 1471-2105-14-204-S1.pdf]

a)

AUC

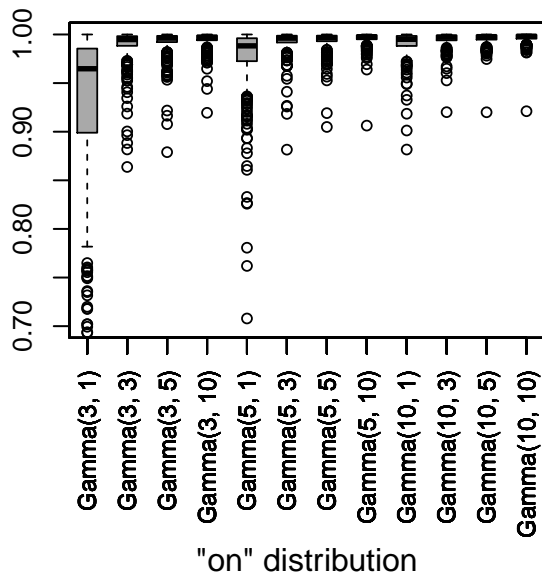**Two lineage patterns**

AUC

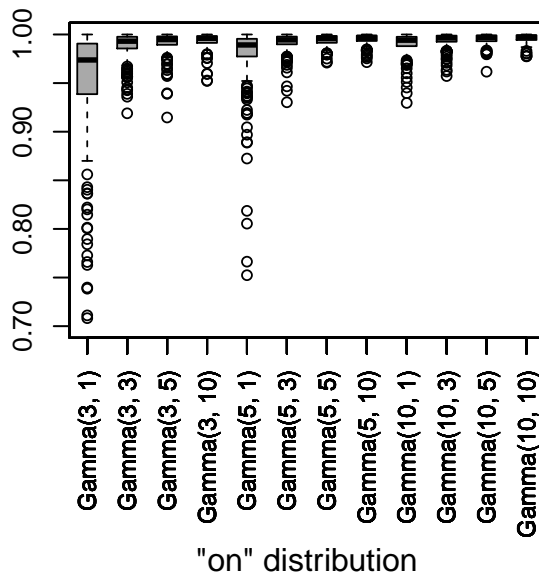

b)

correlation

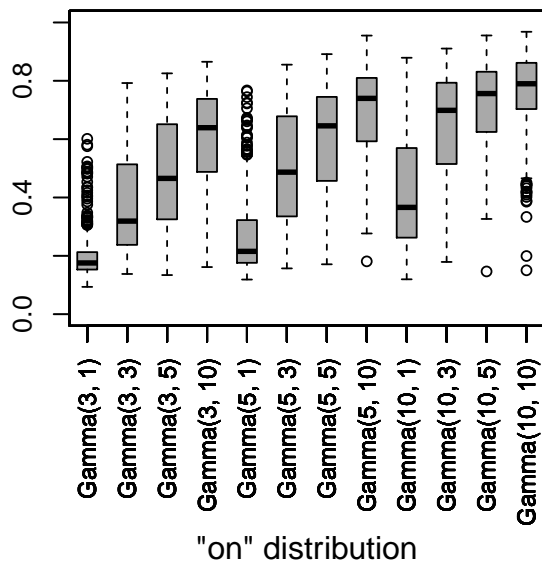**Two lineage patterns**

correlation

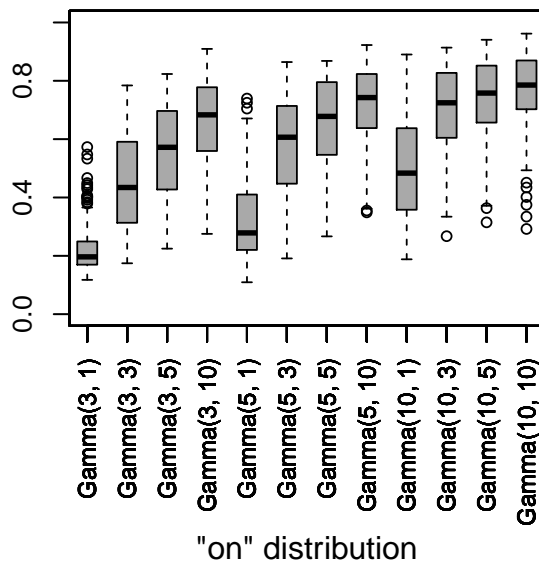

Supplement: Additional file 2: Figure S2 — EP accuracy for one- and two-lineage patterns, measured using a) AUC or b) correlation. Thirty sorted fractions were used. The “off” distribution was drawn from a Gamma(1,1) distribution, and the “on” distribution was the gamma distribution with shape and scale shown on the x-axis. [file 1471-2105-14-204-S2.pdf]

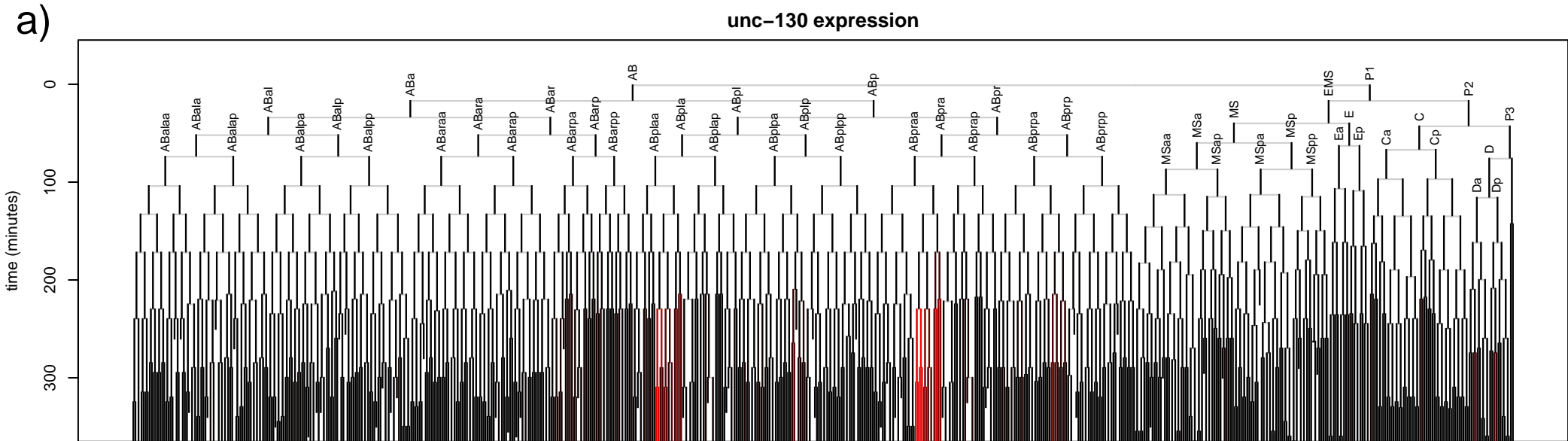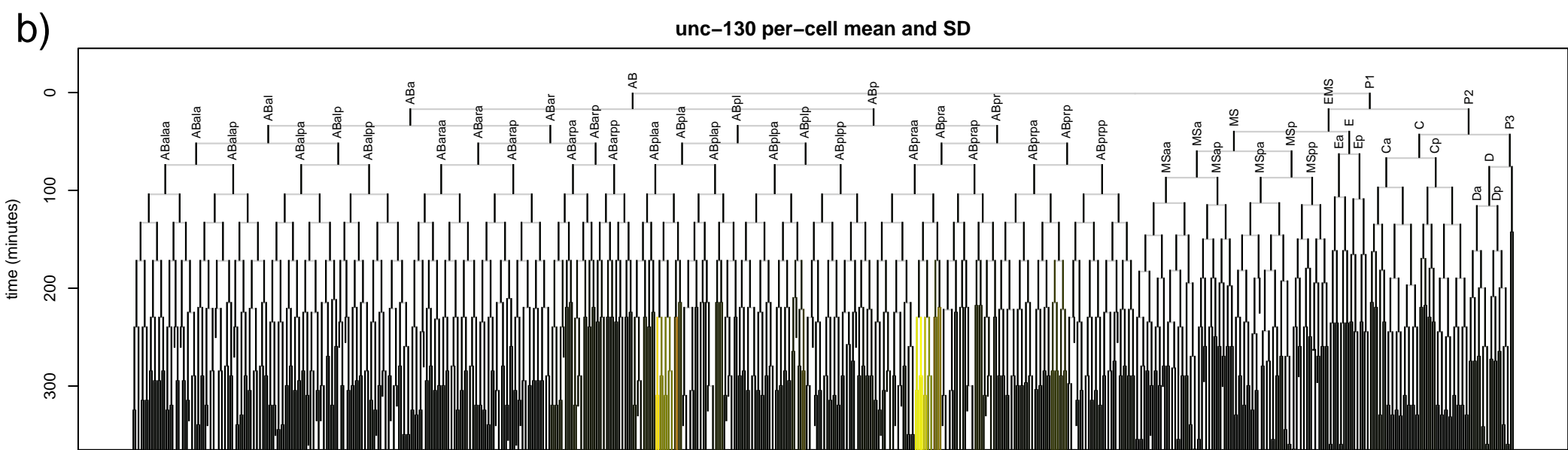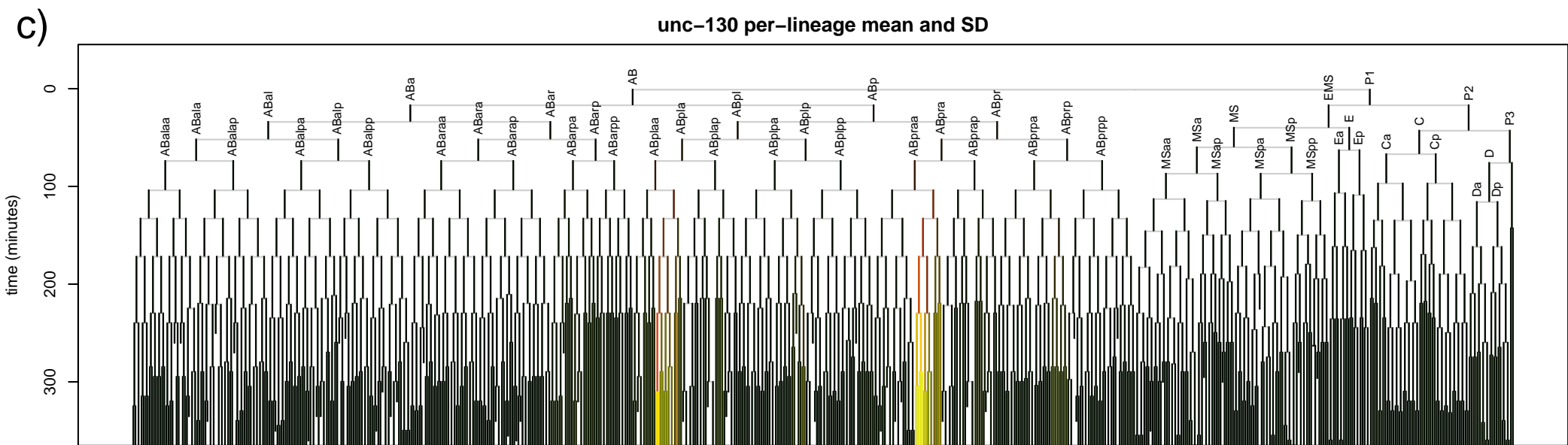

Supplement: Additional file 3: Figure S3 — Prediction bounds for expression of a gene in groups of cells, computed using expectation propagation. Thirty simulated reporters were used. a) Measured expression of unc-130. b) Mean (red) and standard deviation (green) for expression prediction (yellow indicates a large mean and standard deviation.) c) Mean (red) and standard deviation (green) for the average expression in the lineage rooted at a given cell. [file 1471-2105-14-204-S3.pdf]

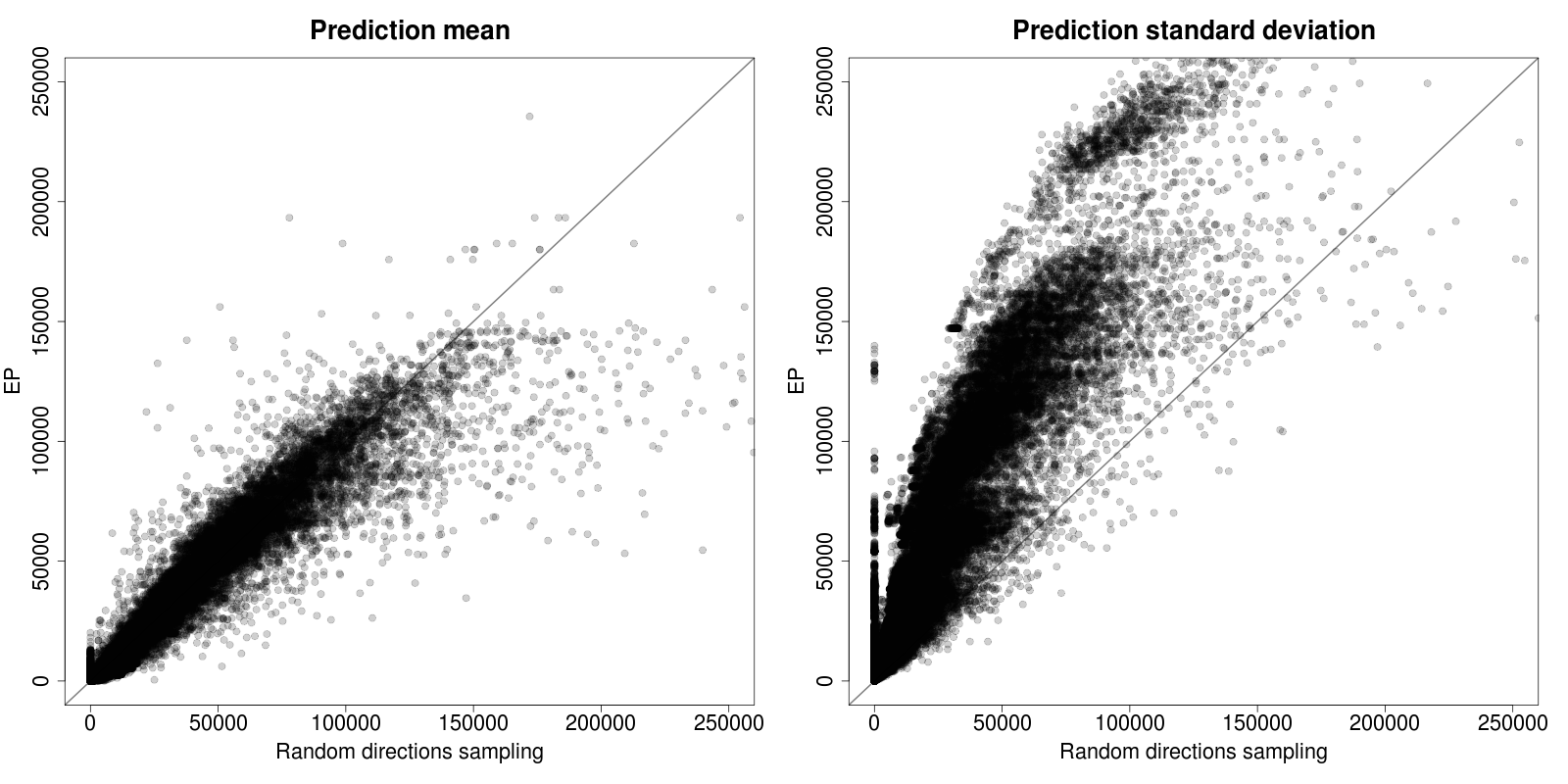

Supplement: Additional file 4: Figure S4 — Comparison of a) mean and b) standard deviation of prediction bounds from sampling and EP, for 123 genes, using thirty simulated reporters. [file 1471-2105-14-204-S4.png]

### alr-1 , 2 million samples

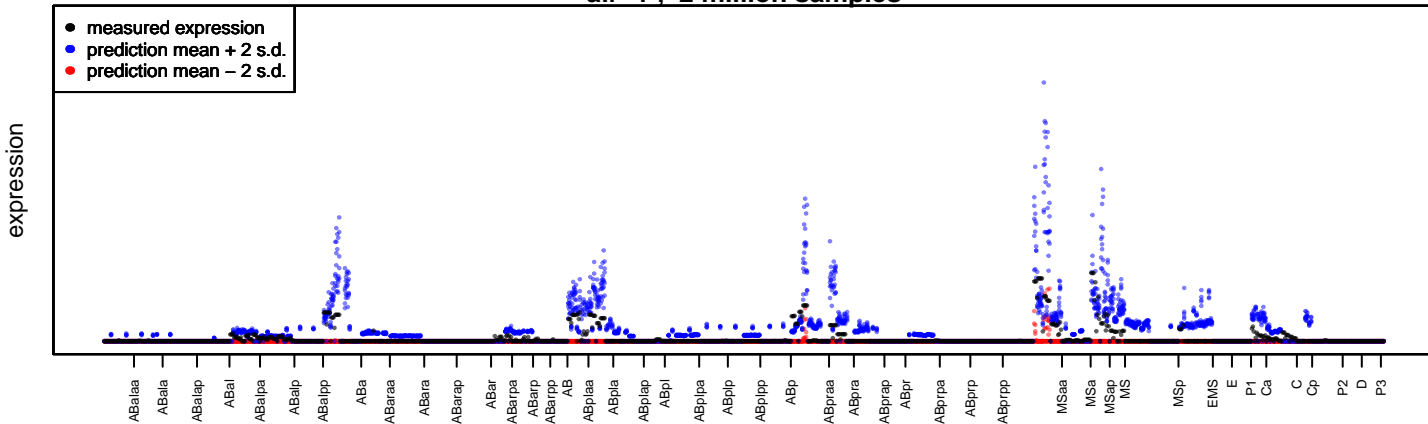

**alr-1 , 10 million samples**

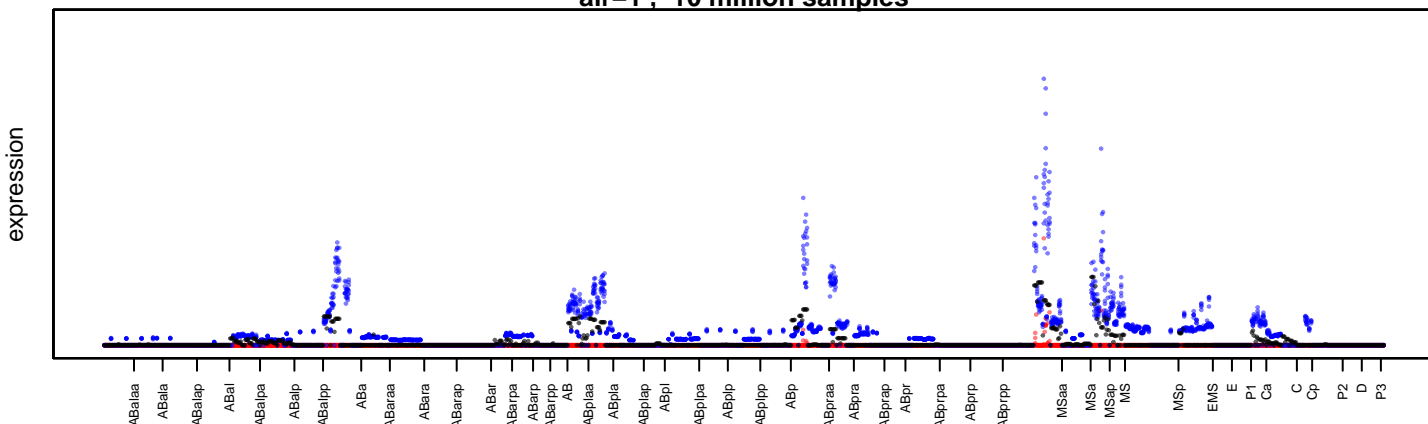

**alr-1 , 20 million samples**

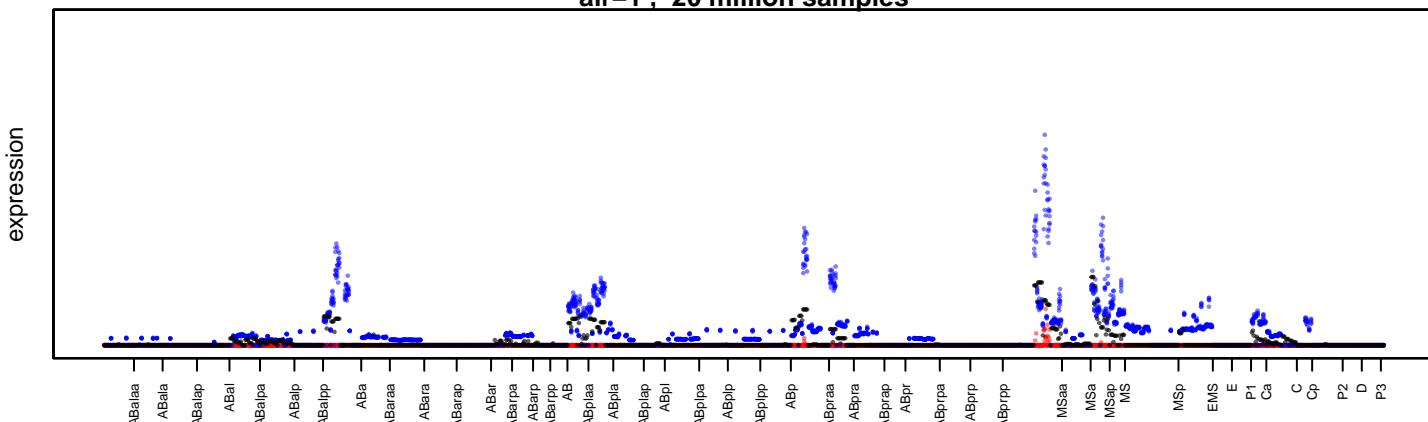

**alr-1 , 50 million samples**

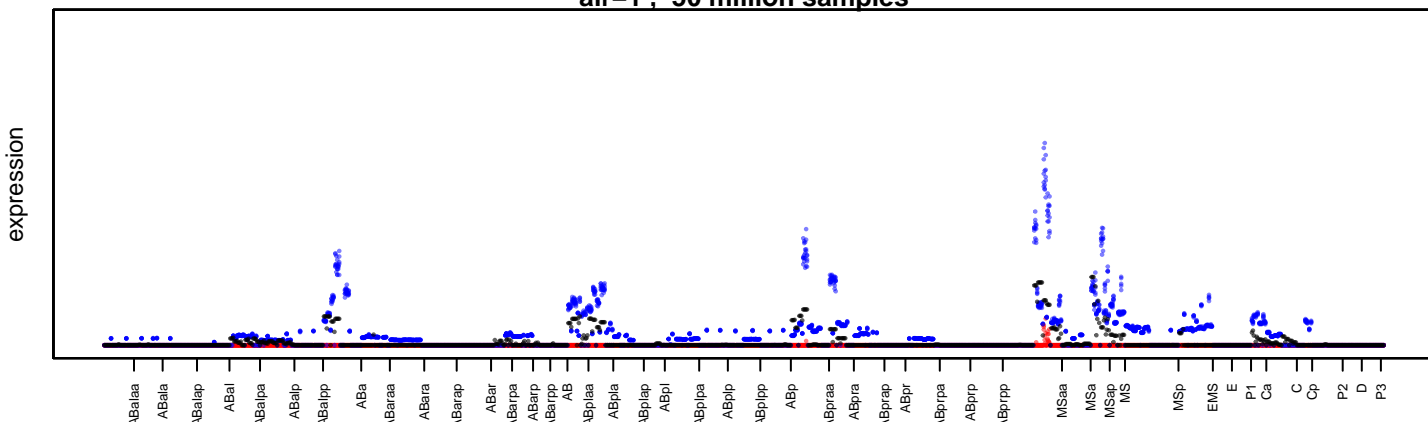

Supplement: Additional file 5: Figure S5 — Two-standard deviation posterior predicted intervals for alr-1, based on mean and variance of increasingly long sampling chains. (Negative values for bounds are truncated at zero). [file 1471-2105-14-204-S5.pdf]

alr-1 potential scale reduction

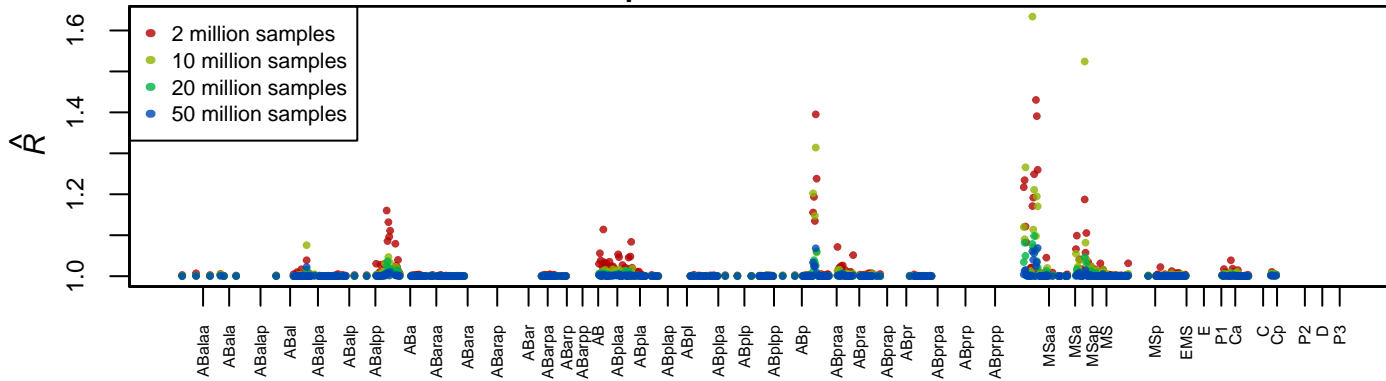

Supplement: Additional file 6: Figure S6 — Potential scale reduction R ([26], pp. 296–298) for alr-1, using increasingly long sampling chains. (Cells whose expression was predicted to be zero by the truncated pseudoinverse method were not included in the sampling, and are not shown). [file 1471-2105-14-204-S6.pdf]
